# A Comparative Analysis of Health Impact Assessment Implementation Models in the Regions of Montérégie (Québec, Canada) and Nouvelle-Aquitaine (France)

**DOI:** 10.3390/ijerph17186558

**Published:** 2020-09-09

**Authors:** Françoise Jabot, Emile Tremblay, Ana Rivadeneyra, Thierno Amadou Diallo, Geneviève Lapointe

**Affiliations:** 1 Univ Rennes, EHESP, CNRS, ARENES–UMR 6051, F-35000 Rennes, France; 2 Direction of environmental health and toxicology, Institut National de Santé Publique du Québec, Quebec City, QC G1V 5B3, Canada; emile.tremblay@inspq.qc.ca (E.T.); genevieve.lapointe@inspq.qc.ca (G.L.); 3 Univ Bordeaux, ISPED, F-33000 Bordeaux, France; ana.rivadeneyra@u-bordeaux.fr; 4 National Collaborating Centre for Healthy Public Policy, Montreal, QC H2P 1E2, Canada; thiernoamadou.diallo@inspq.qc.ca

**Keywords:** health impact assessment, context, municipal level, health promotion, intersectoral collaboration

## Abstract

Many countries have introduced health impact assessment (HIA) at the national, regional, or local levels. In France and in Québec, there is increasing interest in using HIA to inform decision-makers and influence policies, programs, and projects. This paper aims to compare HIA implementation models in two regions: Nouvelle-Aquitaine (France) and Montérégie (Québec, Canada) using a case study methodology. The objective is to gain a better understanding of the similarities and differences in the approaches used to achieve the operationalization of HIA. The methodological approach involves four steps: (1) design of an analytical framework based on the literature; (2) exchanges within the research team and review of documents concerning the two implementation strategies under study; (3) development of the case studies based on the proposed framework; and (4) cross-comparison analysis of the case studies. The findings show that the two regions share certain similarities, including the strong commitment and political will of the public health organizations involved and a well-established culture of engaging in intersectoral action with municipal partners. Differences mainly concern their different approaches to implementing HIAs in accordance with the regional policies and the organizational and administrative contexts in place. This study identifies potential avenues for supporting the practice of HIA at the municipal level.

## 1. Introduction

Health impact assessment (HIA) aims to identify the potential health impacts of proposed policies, programs, and projects before they are implemented and to suggest evidence-based recommendations to mitigate anticipated negative impacts and to maximize positive impacts [1]. While HIA may be undertaken at the national, regional, or local level and applied to a large variety of sectors, most documented HIAs concern urban planning projects at the municipal level [2].

HIA belongs to the family of impact assessments including, among others, environmental impact assessment, social impact assessment, and equity impact assessment [3], but its practice is characterized by a set of distinctive principles and values [4]. It conceives health as a complex and dynamic process resulting from the interaction of environmental, social, and economic determinants shaping population health. HIA places particular emphasis on the unequal distribution of impacts within the population. It is conducted prospectively and involves several stages in accordance with internationally adopted standards and integrates data from the scientific literature and contextual information provided by professionals and citizen experts. Its ultimate goal is to maximize the potential health benefits of the examined policy, program, or project and to advance health equity [5].

HIA traces its origin back to the late 1980s [6] and results from the convergence of concerns in the fields of environmental health and health promotion, notably in Europe and North America. Since then, its practice has gained popularity throughout the world, and in some countries, it has been routinely incorporated in decision-making processes through legal or policy mandates. HIA can be conducted as a stand-alone procedure or integrated in other impact assessments [7]. Practice models vary across countries according to the specific contexts in which HIA evolves and according to the sectors of application (agriculture, transport, urban design, etc.), decision-making levels (local, regional, national), and applied frameworks and methods [8,9,10].

### 1.1. Health Impact Assessment in the Province of Québec and in France

HIA practice has been evolving in Québec since the early 2000s, while in France it has developed more recently, over the past ten years.

#### 1.1.1. A Gradual Emergence in Québec

In 2001, the Government of Québec adopted its Public Health Act. Section 54 of this Act stipulates that “the Minister is by virtue of his or her office the advisor of the Government on any public health issue. The Minister shall give the other ministers any advice he or she considers advisable for health promotion and the adoption of policies capable of fostering the enhancement of the health and welfare of the population. In the Minister’s capacity as government advisor, the Minister shall be consulted in relation to the development of the measures provided for in an Act or regulation that could have significant impact on the health of the population” (Public Health Act, 2001).

Section 54 paved the way for the implementation of an approach inspired by environmental impact assessment (EIA). The Department of health and social services [Ministère de la santé et des services sociaux (MSSS)] is responsible for the new approach, whose aim is to assess the health impacts of legislative provisions and regulations proposed by the Québec government. Although the operationalization of section 54 is inspired by the EIA approach, the context in which the latter is carried out is not, however, applicable to HIA implementation, which has instead given way to a pared down analysis mechanism. Two decades later, the benefits associated with section 54 have been demonstrated and have included, in particular, raising awareness among other departments of the possible effects of their actions on the health of the population. This awareness has also promoted communication, outreach, and legitimization of health issues within certain departments, thus facilitating the consideration of health in public policy development processes [11]. Section 54, however, confers no authority over the decision-making processes of governing bodies external to the provincial Government of Québec, such as municipalities. To develop an area of practice about nonmandatory HIA at the municipal level, the MSSS used and supported the development of expertise in healthy public policy, notably from the National institute of public health [Institut national de santé publique du Québec (INSPQ)] and the National Collaborating Centre for Healthy Public Policy (NCCHPP).

In the wake of section 54, the Montérégie Public health department (PHD) [Direction de santé publique] undertook in 2007 an HIA pilot project at the municipal level and began providing HIA services as part of its regular service offering in 2009 [12]. In 2014, a partnership between Laval University’s Land Management and Regional Planning Graduate School and Québec City’s municipal administration, which joined forces with the Regional Public Health Department, enabled the implementation of the first HIA for the region. Since then, several other HIAs have been completed in Québec City [13]. More recently, the Québec adopted its first Government Policy of Prevention in Health (GPPH). Inspired in part by the Montérégie HIA experience, the 2017–2021 action plan for this policy provides financial support to assist the Regional Public Health Departments in the implementation of HIA in municipal settings. The INSPQ was given a mandate to provide scientific support for the implementation of HIA practice in the eighteen health regions of the province. During the first two years of the action plan’s implementation, it funded and supported sixteen HIAs in fifteen regions. The GPPH action plan also calls for an evaluation of the HIAs completed aimed at determining whether they have strengthened intersectoral action and fostered knowledge sharing between public health actors and municipal decision-makers.

#### 1.1.2. A Growing Practice in France

HIA practice in France is more recent but it is rapidly spreading [14]. The very first instance was an HIA conducted in 2008 at the initiative of a health promotion and sustainable development association closely linked to the WHO European Healthy Cities Network. In 2010, the Ministry of Health organized a national seminar to raise HIA awareness among decision-makers. Since then, HIA practice has grown in France, mainly at the municipal level. In 2013, the National institute for prevention and health education [Institut national de prévention et d’éducation pour la santé] provided funding for a pilot project focused on a public transport infrastructure scheme in the greater Paris region. It also funded a series of training courses whose formats varied in terms of content, duration, and audiences (elected officials, professionals, and practitioners), and, in 2015, it launched a call for proposals for funding three intermediate HIAs. Progressively, HIA has been included as a subject of discussion in conferences and colloquiums, and meetings have been organized to share experiences and promote its practice [14].

More recently, in response to regulatory developments in EIA, a national group mandated by the Ministry of Health has been working on the production of a guide to support the inclusion of health considerations in EIA. In addition, the high council for public health [Haut Conseil de la santé publique] has published a report presenting existing methods and tools for assessing the health impacts of urban development projects [15]. Moreover, the regional health agencies (RHAs) [Agences régionales de santé] have integrated HIA into their health and environmental health programs, placing particular emphasis on urban planning schemes. These developments show that HIA in France is closely linked to healthy urban planning (HUP) and explain why two thirds of documented experiences concern urban development projects. It is also worth noting that there has been enhanced collaboration between the RHAs and the local authorities since 2009 as a result of a territorial reform that created joint local policy schemes (“local health contracts”) aimed at improving health and reducing health inequalities in the territory. Taking advantage of this opportunity and convinced of its potential to place health on political agendas, the RHAs are actively promoting HIA among municipal authorities.

Today, 11 out of 18 French regions have launched at least 1 HIA. Still, HIA practice differs considerably across regions, with the number of HIAs accomplished ranging from 1 to 20. While around 60 HIAs have been launched over the last 12 years, half of them ended at the screening stage or, having been initiated at a very early stage of project formulation, they evolved into a HUP procedure. HIAs are mostly funded by RHAs and conducted by health observatories, private firms and consultants, universities, and, occasionally, municipalities. HIA practice is quite diverse and depends upon RHAs’ awareness, commitment, and understanding of what HIA is and what can be expected. Three RHAs have a particularly proactive approach and have launched evaluation procedures to assess its added value before deciding on large-scale implementation. While there is no national framework guiding HIA practice in France, a small group of practitioners has recently set up a community of practice supported by a web platform created in 2018. This community is creating new opportunities for meeting and sharing experiences and tools during seminars and workshops.

### 1.2. Main Theme and Study Goal

HIA follows a stepwise approach well described in international guidelines and manuals [16,17,18]. However, the ways in which its values, principles, and methodological approaches are implemented and integrated in the decision-making process depend on a variety of factors [7,9,19]. Some factors relate to stakeholders’ commitment, ownership, and capacity to remove barriers between sectors [20,21,22]. Others are linked to the implementation process and, more particularly, to the degree of HIA integration in decision-making processes and to the nature of the pending decision [21,23]. Some authors also point to the overall context, including here the political setting, the prevailing regulatory, administrative and organizational frameworks, the degree of health leadership, and the prevailing evaluation culture, all of which have the potential to become facilitators of or, conversely, barriers to HIA implementation [21,23,24,25]. With regard to this, the recent work of Linzalone et al. [19] analyzing barriers to and opportunities for HIA implementation in different countries provides interesting insights. It places particular importance on two main drivers, political will and legal and administrative frameworks, which activate a “cascade mechanism” triggering other key factors for HIA development, including capacities and expertise, intersectoral coordination, a dedicated structure or an HIA champion, and funding. Finally, other authors have identified the growing interest in integrating health in other forms of impact assessment such as EIAs as a key factor for HIA development [24,25,26,27,28,29].

These findings led us to postulate that differences and similarities of HIA practice in Québec and in France could be largely attributed to their specific decision-making, administrative, and organizational environments. In order to explore how these environments influence HIA emergence and progress, we conducted a comparative study of two selected regions, Montérégie in Québec and Nouvelle-Aquitaine (NA) in France, both of which benefit from a regional implementation scheme launched by the health authorities and targeting municipalities. The study goal was to explore the contextual conditions and mechanisms enabling HIA development and to provide lessons learned that could benefit public authorities in the two regions and in other jurisdictions interested in promoting HIA at the municipal level.

## 2. Materials and Methods

The present study was led by an international team of five public health researchers and practitioners involved in a number of HIAs in Québec and in France. A case study methodology was developed for gathering, analyzing, and comparing data from the two regions whose implementation models are the subject of the case studies. Well aligned with the study goal, this methodology has proven to be particularly appropriate for exploring a phenomenon within its real-life context and for analyzing its interactions with a number of factors relevant to the research question [30].

A four-phase research process was used. First, an analytical framework was developed based on a literature review of existing frameworks for describing key contextual factors, preconditions, and mechanisms enabling HIA implementation [2,7,19,21,31,32,33], more precisely: (1) contextual factors: political support and commitment, policy or administrative framework, legal mandate; (2) implementation scheme: stewardship, roles and responsibilities, technical support and expertise, HIA model, and purpose; and (3) HIA delivery: proponents, practitioners’ profile and capacities, type of HIA, decision level, sector of application, funding, capacity building, and resource generation. Second, in order to ensure that the specificities of the two regional contexts were taken into account, exchanges within the research team and a review of documents related to the implementation schemes in the two regions were undertaken. These inputs added new dimensions to the original analytical framework and substantiated those found in the literature. Third, the two case studies were developed with a particular emphasis on capturing key dimensions related to the regional policy and administrative contexts in place. Finally, a cross-comparison analysis of the similarities and differences of the two implementation models was conducted based on the main contextual factors identified by Linzalone [19].

Data used for developing the case studies was gathered through content analysis of documents and reports describing the two implementation schemes, informal interviews with key stakeholders (regional and municipal policy-makers, departmental officials and technical staff, public health officials, HIA consultants, and practitioners), and direct observations during seminars, workshops, and other HIA-related meetings held in the two regions.

According to Linzalone [19], the existence of policy and legal frameworks are a prerequisite for activation of the key mechanisms facilitating HIA implementation: capacity building and expertise, provision of resources, and intersectoral coordination. For the purposes of this study, we adopted Lacouture’s definition of mechanism [34], which describes a mechanism as that which “results in the interaction between human agents, intervention and structures” and occurs in a given context.

The cases studies were developed in accordance with the aforementioned analytical framework.

The cross-comparison analysis of the two implementation models was based on the following dimensions: (1) organizational framework (HIA integration into existing plans, policies, and programs; coordination of health and environment sectors; and dedicated unit and funding); (2) capacity building (methodological tools and procedures, training, external support, and setting up a reference structure); and (3) governance: leadership, sharing of roles and responsibilities; arbitration among antagonistic expectations and interests, and decision-making process.

## 3. Results

### 3.1. HIA Implementation Model in Montérégie

HIA began in Montérégie with a pilot project in 2007–2008 when the first stages of the process were applied to three municipal projects. The evaluation of this experience highlighted the feasibility, acceptability, and relevance of HIA for public health actors and municipal decision-makers [12]. The conclusion drawn from this experience led the PHD to include HIA in its 2009–2012 regional public health action plan (RPHAP), and to conduct its first complete HIA in 2010–2011. The integration of HIA into the RPHAP, with the objective of promoting healthy public policy, made it possible to give HIA a formal status and dedicate resources to it. HIA is now a free service offered to municipalities upon request. Over the past ten years, some 20 HIAs have been conducted in the region on both urban development projects and social policies. Two evaluations of HIA implementation in Montérégie carried out during this period also made it possible to validate or refine certain practices. To manage the recurrent implementation of HIA, the PHD established in 2011 an approach adapted to the Québec context aimed at satisfying the requirements of HIA practice, meeting the needs of municipalities and ensuring coordination of stakeholders. This approach relies on regional and local public health teams to conduct the HIA process while ensuring the integration of municipal partners at each stage of the HIA.

**Figure 1:**
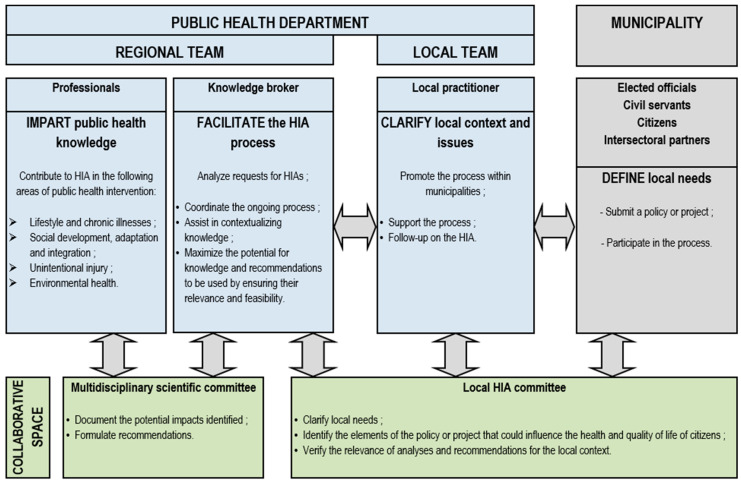
Health impact assessment (HIA) implementation scheme in Montérégie.

The approach developed brings together four categories of actors, namely a knowledge broker, public health professionals with topic-specific expertise, a local public health worker, and the municipal partners responsible for planning or executing the project subjected to the HIA. Each of these actors is invited to sit on either the local or the scientific committee set up for each HIA, with the exception of the knowledge broker who participates in both committees. The Figure 1 below shows the roles each actor is expected to perform and their respective contributions to the local or scientific committees. According to this approach, the knowledge broker is responsible for leading the process and coordinating all the tasks necessary for the proper conducting of the HIA. Various professionals from the PHD are selected and then invited to participate in the HIA based on the issues raised by the municipal project and the expertise they possess. A professional from the local public health team joins the process to provide insight into the local context and point toward issues to be considered during the HIA. The municipal decision makers are not entitled to realize the HIA but have an active role to play in it. These partners are invited to present the project with as much detail and transparency as possible and to share any documents or information likely to inform the analyses. During the process, the decision makers are also invited to comment analysis and recommendations to make sure they fit in the project’s context. Any relevant suggestions are considered to adjust the final report.

The local HIA committee provides a space for interaction between municipal partners, the local public health actor, and the knowledge broker. This committee aims to provide the conditions conducive to the implementation of the HIA’s steps, which requires the involvement of municipal partners in activities such as the description of the project, the selection of the elements of the project that are to be analyzed, as well as the validation of the accuracy of the analyses and the feasibility of the recommendations. The multidisciplinary scientific committee includes the PHD professionals selected for an HIA and the knowledge broker. This committee is the locus for sharing all the information necessary for the appraisal of the potential impacts of the project and the formulation of recommendations. As the knowledge broker is the only person who sits on both committees, he or she becomes responsible for facilitating them and sharing information among stakeholders.

Regardless of the municipality or the complexity of its project, HIAs in Montérégie are a free public service, as are other public health services. Even if the estimated cost of an HIA is between $30,000–40,000 depending on the level of complexity and depth of the HIA, the PHD funds HIA services from its regular budget, which means that it receives no additional funding for their implementation. Because the conducting of the HIA process and the production of scientific analyses draw on the internal resources of the PHD, the team’s average production capacity is two intermediate HIAs per year, for a region of 1.2 million people and 147 municipalities. The training of resources is conducted gradually when new members are integrated into the PHD teams and when they participate in an HIA. The tools used are created by the teams or are adaptations of tools borrowed from other organizations.

### 3.2. HIA Implementation Model in Nouvelle-Aquitaine

HIA was introduced in Nouvelle-Aquitaine as a result of the RHA director’s commitment to healthy public policy coupled with the internal advocacy work conducted by a public health actor, which led to the launching of three HIA pilot projects in 2015. The first one was designed as a pilot project intended to assess HIA potential and to develop local capacities. Subsequently, the RHA initiated a policy intended to actively promote HIA development. The RHA’s territorial departments were assigned the task of ensuring the accomplishment of two HIAs per year in collaboration with the municipalities within their circumscription. For this purpose, dedicated funding was allocated and HIA was included in the Regional Environmental Health Program (REHP) 2017–2021, which identified it as an effective measure for achieving healthy environments.

**Figure 2:**
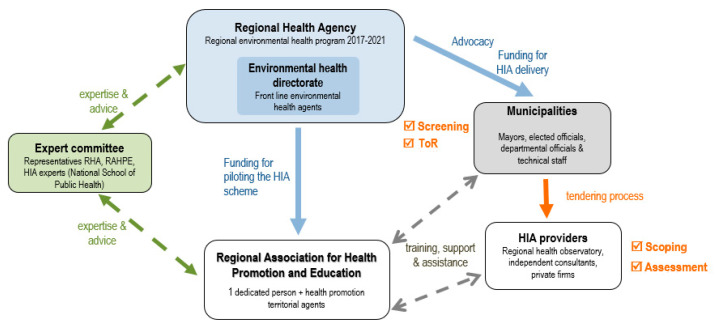
HIA implementation scheme in Nouvelle-Aquitaine.

As shown in Figure 2, the implementation scheme brings together three main actors: the RHA (represented by the Environmental Health directorate), the municipalities, and the Regional Association for Health Promotion and Education (RAHPE) [Instance régionale d’éducation et de promotion de la santé]. The latter is assigned the mandate of coordinating and piloting the scheme. In addition, a technical committee integrating representatives of the RHA, the RAHPE, and several HIA experts meets at the request of the RAHPE and provides advice on validated tools, quality standards, and the overall functioning of the scheme. The RAHPE is also responsible for developing HIA awareness campaigns among municipalities, organizing training sessions, disseminating adapted tools and procedures, and providing technical support to HIA practitioners. The implementation scheme has a budget allocation of € 540,000 for 2017–2021, out of which € 203,000 are allocated to the RAHPE’s piloting mandate and € 357,000 to municipalities responsible for commissioning HIAs (€ 30,000 per department). HIAs are applied to urban development projects proposed by the municipalities. To date, twenty HIAs have been initiated in 10 of the 12 departments, most of them conducted by the regional health observatory [Observatoire régional de santé] or by independent consultants and private firms. Just over half of them have been completed and a report is available. They are essentially intermediate, stand-alone HIAs and follow the stepwise procedure proposed in international guidelines and manuals.

Every HIA is preceded by advocacy work conducted by the RHA territorial agents, supported by the RAHPE, and aimed at supporting elected officials in identifying a municipal project that could benefit from an HIA procedure. Once the project has been selected, the municipality launches a public tender process to solicit HIA providers. A one- to two-day training session targeting all stakeholders is conducted at the scoping stage to establish a shared culture informed by HIA values. The advocacy approach targeting municipal actors and this multi-institutional training session constitute the main levers for promoting HIA development throughout the region. While the RAHPE is responsible for providing technical support and assistance to ensure compliance with HIA quality standards and procedures, the diversity of practitioners’ profiles in terms of HIA-related knowledge and experience makes HIA practice quite heterogeneous. Some HIAs only adhere very partially to practice standards and, bearing a closer resemblance to other procedures, such as community participation or public consultation processes, they do not fully qualify as HIAs. The RAHPE is aware of this and, as advised by the technical committee, is currently refining established procedures and tools and reviewing practices at regional meetings with the main stakeholders. After three years of operation, there is growing concern about the need to evaluate the implementation scheme and to strengthen governance at the regional level.

### 3.3. Cross-Comparison Analysis of the Two Implementation Models

The two-implementation schemes present some similarities, particularly in terms of prevailing political commitment, but differ in certain respects and notably in their governance models. Table 1 below presents the main features of the two implementation models.

HIA in Montérégie and Nouvelle-Aquitaine is undergoing thriving development at the municipal level as a result of the strong political will of regional and local decision-makers. These two actors share a commitment to healthy public policies and subscribe to HIA principles and values, namely, a holistic approach to health, a concern for greater equity, and a willingness to fulfill citizens’ expectations. HIA practice in the two regions share several other features: (1) it is included in regional and/or local planning instruments, is undertaken on a voluntary basis as a stand-alone procedure, and is aimed at informing the decision-making process; (2) HIAs are mainly applied to urban development projects and are carried out in close collaboration with the municipal authorities responsible for the project and in accordance with internationally adopted standards; (3) they include a preliminary stage in which the regional health institutions conduct HIA advocacy among municipal decision-makers; and (4) expert advice and adapted tools are made available in both regions to support local teams conducting HIAs.

There are also major differences influencing HIA practice in the two regions, the main one being their respective piloting models. Whereas, in Montérégie, responsibility for conducting HIAs falls to the PHD, in Nouvelle-Aquitaine, several actors share responsibility throughout the HIA process, with external providers taking charge of carrying out the data collection and impact assessment and making recommendations. As shown in Figure 1 and Figure 2, certain stakeholders are involved in one model and not in the other (external service providers in Nouvelle-Aquitaine, knowledge brokers in Montérégie), and the sharing of responsibilities among stakeholders varies. In Montérégie, it is the public health team at the PHD that carries out HIA procedures at every stage of the process, whereas in Nouvelle-Aquitaine, the RHA actors are only involved at the very beginning, as impact analysis and recommendations are entrusted to external HIA practitioners. Moreover, while the knowledge broker in Montérégie is actively engaged and ensures continuity throughout the process, the intervention of the RAHPE in Nouvelle-Aquitaine is not systematic and varies from one territory to another depending on the requests made by municipalities. In terms of training, newly recruited public health professionals in Montérégie acquire skills and competencies as they are integrated into the experienced PHD team responsible for conducing HIAs. In Nouvelle-Aquitaine, HIA providers have diverse profiles in terms of HIA knowledge and experience, even though they all participate in the training session organized at the scoping stage to establish a shared culture informed by HIA values. Finally, the two regions differ with respect to the mandate assigned to the regional actors: in Montérégie, the number of HIAs carried out is determined by the capacity of the PHD team carrying out the HIAs, while in Nouvelle-Aquitaine the territorial departments are expected to accomplish a set number of HIAs annually.

## 4. Discussion

The comparison of the two-implementation models reveals organizational differences impacting governance modes. For the purposes of this study, governance refers to the specific logic and processes regulating relations between professionals, institutions, and civil society, coordinating actions and arbitrating choices with a view to a negotiated decision. Thus, as noted by Meuleman [35] “each governance environment (…) affects the governance of impact assessments.” These assertions provide some clues as to how to discuss the influence of governance styles on approaches to HIA implementation, on the reasons for choosing certain options, and, more broadly, on how HIA practice in the two regions fits within the context of other international experiences.

### 4.1. Influence of Governance Modes on Approach to HIA Implementation

HIA implementation involves a plurality of actors of varying status and profile acting at different levels of intervention in accordance with the established framework for sharing responsibilities and the associated procedures facilitating exchanges and following a timeline for the planned activities. These organizational features impact the operation of the HIA scheme in terms of its effective implementation, stakeholders’ commitment, and HIA practices.

#### 4.1.1. Leadership and Sharing of Roles and Responsibilities

While in Montérégie, both leadership and funding are provided by the PHD, the implementation scheme in Nouvelle Aquitaine is based on a continuum of actors with interwoven tasks taken up as the HIA stages progress. The screening stage is the responsibility of the RHA and the RAHPE territorial agents, who are not always sufficiently equipped to establish the value of conducting an HIA. Following screening and project selection, the municipality prepares the terms of reference and selects the HIA provider. Even when embarking on its first HIA experience, the municipality does not always seek support from the RAHPE, which is responsible for supporting the local actors conducting HIAs. The municipality has no obligation in this respect, and the RAHPE has no real power of influence, either over the scoping stage or over the selection of the HIA provider. Furthermore, once funding is allocated to the municipality, the RHA is no longer expected to intervene. This allows the municipality to manage the launching of the procedure on its own until an external HIA consultant is hired. A privileged relationship is then established between the consultant and the municipality, and the other stakeholders are not reintegrated until the end of the process. While the implementation scheme is based on the involvement of three institutional pillars (the RHA, the municipalities, and the piloting structure) and on the integration of environmental and health promotion approaches within the local HIA team and the piloting structures, in practice, the segmentation of activities across a multiplicity of actors creates areas of autonomy for the municipality and the HIA provider, who are often inexperienced, which can weaken the coherence of the whole procedure. Although balanced and complete, this scheme would require the designation of a true piloting entity to operate throughout the HIA process. The Montérégie scheme, where the PHD cumulates responsibilities, including funding, coordination, training, and implementation, undoubtedly avoids potential misunderstandings regarding stakeholders’ roles and responsibilities. Here, the municipality is instead the beneficiary of the HIA, although it can play an active role in its achievement. The fact that leadership is shared between the PHD directorate and the knowledge broker ensures sustainability and an iterative practice that builds on previous HIA experiences. The implementation procedure resulting from the cumulative experience of more than ten years facilitates a clear definition of roles and responsibilities across stakeholders throughout all HIA stages. This steady development of the HIA procedure has allowed the PHD team to better adapt to the organizational and legislative context of Québec municipalities, to facilitate the acculturation of public health actors to local issues, and to adapt its language to municipal realities.

#### 4.1.2. Degree of Stakeholder Incentivization and Buy-in

The HIA implementation policy in Nouvelle-Aquitaine reflects the determination of the RHA director, who set an ambitious objective for the territorial departments in terms of HIAs to be accomplished annually. However, this voluntarist objective encounters two pitfalls: the variable receptiveness of the municipalities and the degree of acceptance by the RHA territorial agents responsible for carrying out HIA advocacy and supporting the municipalities. Indeed, securing the commitment of the municipal authorities requires building awareness and understanding of how HIA can contribute to their agendas and its added value. The time required for effective acculturation to HIA values and principles is hardly compatible with the number of HIAs to be conducted annually and undermines success. In addition, the RHA territorial agents are not well acquainted with health promotion approaches and do not espouse the HIA advocacy role they have been assigned or, more broadly, a mission they perceive to be poorly defined. Although the challenge of convincing municipal actors of the interest of HIA is also present in Montérégie, because they have a background in health promotion, the PHD professionals adhere more easily to HIA values and objectives and they are better able to conduct their advocacy mission among municipal decision-makers. It should also be noted that in Montérégie no quantified annual objective has been established, and the PHD’s mission is focused instead on supporting healthy public policies.

#### 4.1.3. Resource Allocation and Capacity Building

The inclusion of HIA within the PHD service portfolio in Montérégie, in addition to providing legitimacy, facilitates the allocation of human resources and helps entrench the procedure. Because training and support are limited to a few guides and resource persons, the development of HIA professional expertise relies strongly on a learning by doing approach. In addition, the skills developed within the PHD’s HIA team benefit all the HIAs conducted, as well as other health promotion activities. In Nouvelle-Aquitaine, while the RHA territorial agents have benefited from brief training in HIA, their environmental health culture is far removed from the health promotion approach required to fully capture the HIA approach and its contribution. Very often, depending on their professional experience and their degree of collaboration with their RAHPE teammates, they find themselves lacking both the competency and the legitimacy to carry out a mandate for which they do not consider themselves sufficiently trained.

#### 4.1.4. Diversity of HIA Practitioners’ Profiles and Potential for Innovation

HIA teams in Montérégie have been engaged in the implementation scheme from the beginning. They have been confronted with a variety of situations, acquired solid practice skills and competencies, developed guides and tools, and refined and standardized their methods. In addition to improving efficiency and consolidating the HIA process, the newly acquired professional skills have benefited other health promotion activities. In Nouvelle-Aquitaine, municipalities use external HIA providers with varying profiles and levels of experience. Although such diversification can lead to heterogeneous HIA practice, this can prove to be an asset by driving HIA progress and encouraging methodological innovations. Moreover, the recent organization of dedicated meetings in the twelve territories of the region has enabled productive exchanges of know-how among HIA practitioners and stimulated innovation.

### 4.2. Two Implementation Schemes Inserted in Distinctive Contexts

HIA practice takes place in specific political, institutional, and administrative contexts. Prevailing management traditions and the logic and culture of actors in the two regions examined may help explain the differences found in the two implementation models under study.

In both Montérégie and Nouvelle-Aquitaine, HIA development was a response to regional health authorities’ interest in using HIA to strengthen their partnership with the municipalities, to develop intersectoral action and to advance healthy public policies. Nevertheless, cumulative experience with HIA, its adoption by health institutions, and its integration with prevailing policies and structures led to different forms of organization in the two regions.

#### 4.2.1. Pre-Existing Structural Context

In Québec, the adoption in 2001 of the Public Health Act and its section 54 definitely helped promote the concept of healthy public policies. This concept has been differently operationalized across regions in Quebec. In Montérégie, it has certainly led to HIA implementation at the municipal level but also to broad reflection on the topic and the implementation of intersectoral actions. The positive evaluation of the first HIAs, both by municipal partners and the PHD, has contributed to perpetuating its practice. HIA was included in the Regional Action Plan 2009–2016 and again reintroduced in the new plan 2016–2020. The Montérégie experience and the effectiveness of HIA at the municipal level encouraged the Québec government to integrate it into the government’s first preventive health policy (GPPH) in 2016. Its action plan proposes an HIA development support program aligned with the Montérégie implementation model, that is, based on a governance model in which the PHD assumes a coordinating role and carries out HIAs.

HIA emergence in France is more recent, and its development was not preceded by national reflection nor was it linked to a regulatory framework. Nevertheless, information about the first HIA experiences spread from one region to another during meetings and training sessions. The introduction of HIA in Nouvelle-Aquitaine is part of a series of events arising from the combination of a national climate favorable to HUP approaches, the existence of planning instruments made available to the regional health authorities and the municipalities, and the internal advocacy work conducted by a public health professional who had the ear of the RHA’s director. Following the success of the first pilot projects, the RHA opted for a policy of large-scale implementation by setting the number of HIAs to be accomplished annually in each territory of the region. However, the lack of sufficiently trained staff within the RHA required to achieve this ambitious goal led to the outsourcing of the execution of HIAs and the coordination of activities on the territory. It should also be noted that, with the territorial reform of 2015, Nouvelle-Aquitaine integrated three former regions and became the largest region in France. Its vast territory and the fusion of three previous RHAs, not all located in the regional capital, have shaped current relations between the regional and the territorial levels. Within this configuration, delegating the piloting function to an external health promotion structure with local agents throughout the territory should enable effective coordination of activities and stakeholders as well as close monitoring of the implementation scheme.

#### 4.2.2. HIA Approaches and Underlying Representations

Prevailing representations of HIA, concerning, namely, HIA’s nature (evaluation study, intersectoral instrument, strategic tool, etc.), HIA’s function (producing knowledge, improving a project, informing public policies, empowering citizens, etc.), and HIA’s integration within institutions (one activity among others, an intervention principle applied to several policies, etc.) have also shaped the implementation models, particularly in operational terms.

In Québec, HIA is conceived of as a means of achieving healthy public policies and it is part of an active policy of promoting intersectoral action between the public health sector and the municipalities. The knowledge broker’s mission is to enable the exchange and adaptation of scientific knowledge facilitating the consideration of HIA recommendations and to promote a better use of evidence by municipal decision-makers [36]. The aim of establishing a partnership between public health actors and municipalities is to make useful and relevant knowledge available to decision-makers in the context of their project development processes.

In contrast, HIA in Nouvelle-Aquitaine is conceived as an outsourced activity with regard to data collection, impact assessment, overall analysis, recommendations, and reporting. Within this configuration, the RHA’s function is limited to promoting and funding the implementation scheme. This is not only the case for HIA but also for other research and evaluation activities (program evaluations, strategic studies, statistical reports, etc.), which are rarely carried out in-house, due to a lack of human resources and because they are not considered priority tasks. Resorting to external consultants or private firms is actually a common practice in France. Moreover, the figure of the knowledge broker does not exist, and the notion of knowledge management and transfer has not been formally introduced [37]. In addition, in the field of public policy evaluation, researchers have questioned for decades the role of the evaluation [38], its institutional implementation, and other challenges related to evaluation typologies (internal or external) [39]. Because of its evaluative nature, HIA is confronted by the same questions: who should conduct HIAs? Public health officers, external consultants, other stakeholders? [40]. Given the close link between HIA and decision making, and the contributions of policy evaluation research, we consider that all these different actors are legitimate to carry out HIAs. Their specific contribution and role will vary accordingly to the context in which HIA practice evolves. Nevertheless, whoever is conducting the HIA, commitment and collaboration between these stakeholders is desired to ensure a balance of views, ownership of results, and HIA effectiveness. To this end, the public health sector has a role to play in mobilizing partners, building bridges between sectors and facilitating the process coordination.

#### 4.2.3. The Stance of the Regional Health Structures

The stance of the regional health authorities is a major issue. In Montérégie, the PHD assumes the leadership of the scheme, whereas the RHA’s stance in Nouvelle-Aquitaine is more ambiguous and stems from the history of relations between the regional authorities and territorial actors in France. Although designated as the highest authority in the field of health, the Central Government has gradually delegated its leadership to other institutions acting at a regional level, notably the territorial authorities. The main challenges confronted by the regional authorities during the past 15 years have included the need to facilitate policies rather than pilot them in an authoritarian manner, to establish intersectoral approaches, and to link national orientations to local policies [41]. Within the HIA context, the RHA must fully play its role as a health policy facilitator assisted by advocacy and supportive actions, while allowing municipalities the autonomy they need to take ownership of the HIA approach. The RHA’s ambiguous stance with respect to the governance of the HIA scheme reflects the need to search for a more participatory mode of regulation capable of legitimizing a process led by the municipalities.

### 4.3. Linkages with International Contexts

International literature reports on HIA implementation around the world [33,42] from historical as well as theoretical, methodological, or even ethical perspectives [43]. While implementation strategies and differences between countries are well documented [7], there are fewer publications on the relationship between impact assessment, of any type, and its governance environment [35,40].

The approaches to HIA implementation in the two case studies analyzed in this article reflect what is observed in many countries. In these two cases, HIA is developed locally and applied to urban policies, as in a majority of countries. It is often integrated into the HiAP approach (Québec) or serves as a basis for developing healthy public policy or more equitable public policies, as is the case in Scotland, Ireland, the Netherlands, Sweden, and Wales [23,44,45]. Elsewhere (Korea, India, Thailand, and the USA), connections are made instead with environmental impact assessment or healthy urban planning (France) [23].

#### 4.3.1. The Driving Role of the Health Sector

In general, the health sector plays a leading role in directing advocacy actions that can prompt the establishment of regulatory frameworks or the launch of HIAs. In Sweden, HIA has benefited from the placing of health and inequality issues on the political agenda [44]. In Denmark, the Ministry of Health first attempted to introduce HIA at the national level in 1996, but the proposal made to the government was not accepted and HIA developed at the local level [23]. This example, as well as that of Montérégie, where local dynamism operates independently of national regulations, clearly demonstrates that the existence of a legal framework, although cited as a factor favoring HIA implementation [23,33] is not always necessary and certainly not sufficient. In New Zealand, local governments responsible for the delivery of public health services have established strong collaborations with public health for the achievement of HIAs with the support of the Public Health Advisory Committee, a body that is politically and administratively independent [23]. In Ireland, HIA was boosted by the publication of a proposal for a National Environmental Health Action Plan, followed by the national health strategy [25]. Finally, the WHO Healthy Cities network has played a catalytic role in many European countries since WHO included HIA in the fourth phase of the action program for the 2004–2008 period [46]. Galway in Ireland and Rennes in France were the first cities not only to join the network but also to initiate and promote HIA [14,25]. These experiences show that HIA development greatly depends on the advocacy function of the health sector and its ability to actively seek opportunities to collaborate with local authorities to promote its practice.

#### 4.3.2. A Plurality of Organizations Fulfilling Several Functions

The organizations that conduct HIAs may be public institutions, including public health institutes, universities, or private consultants, and are sometimes organized into a network. In addition to carrying out HIAs, they perform training and supervisory functions for structures and actors considering innovative ways of implementing HIA [4,23]. In New Zealand, academics developed and delivered training programs, assisted teams, and also conducted assessments of both the process and the impact of HIAs. In Denmark, they intervened at the request of the municipalities to support them in conducting HIAs. They also helped put HIA on the agenda of government authorities and developed robust methods for assessing the process and demonstrating its effectiveness. In the State of New South Wales in Australia, HIA was mainly developed by the Center for Health Equity Training, Research and Evaluation (CHETRE) of the University of New South Wales in partnership with governmental and nongovernmental actors. This centre supports agencies in carrying out HIAs through a learning-by-doing approach. In the United States, several organizations, in the government, academia, nonprofit associations, foundations, and the private sector, contribute to the practice of HIA. Foundations or health institutes such as the Centers for Disease Control and Prevention have been involved in HIA funding. Furthermore, students enrolled in HIA courses have sometimes completed HIAs under the supervision of teachers [2]. In Sweden, actors of varying status (civil servants, municipal officials, experts, and council members) have organized themselves into a network in order to promote the development of HIA and make available training resources useful for conducting HIAs [44]. Finally, in Ireland, the Institute of Public Health provides a set of educational resources, facilitates a forum each year and supports a network of stakeholders. It received a mandate to construct an evidence base by publishing reviews of the literature in certain fields (employment, transport, the built environment) [25]. Québec and France are no exception to countries engaging in these practices. In Québec, the Institute of Public Health has a mission similar to that of the Irish Institute of Public Health. In France, a small number of universities with teachers who have used HIA have introduced it into training courses. The National School of Public Health (École des hautes études en santé publique (EHESP) was the first to become involved in this area by carrying out the first HIAs. It delivers lessons, produces tools, supervises teams, develops research, and coordinates the network of the emerging HIA community.

#### 4.3.3. Effective and Clear Governance

The lack of leadership and the vagueness in the distribution of responsibilities identified in the French case were also found in other countries such as Ireland, where the various agencies and actors from the health and environment sectors and from local institutions that intervene tend to work in silos [25]. This question of regulatory authority is nonetheless decisive for the implementation of intersectoral actions and crucial in the absence of a legal status for HIA. Meuleman [35] recalls the importance of understanding the governance framework, including its traditions and its implicit values, given its influence on impact assessment and by extension on an HIA’s ability to influence the policy process [23].

### 4.4. Limitations of the Study

This article has focused on analyzing HIA implementation approaches in Montérégie and Nouvelle-Aquitaine and formulating explanatory hypotheses regarding the differences observed in terms of HIA operationalization and practice model. The effectiveness of each approach has not been studied due to the lack of comparable evaluative studies in the two regions. Indeed, an evaluation of a series of HIAs was carried out in Montérégie [47], whereas only one HIA was evaluated in Nouvelle-Aquitaine [48]. Moreover, this paper is based on field data and on the reflective analyses of the authors involved in different roles (evaluators, practitioners, and assessors) in the HIA schemes under study. This situation is, on the one hand, an asset, owing to the depth of information drawn from both documentation and from direct observations in various contexts (workshops, training, conducting and/or supervising HIAs, and reflective seminars) but it is also a source of bias as it might compromise distance and impartiality.

## 5. Conclusions

Montérégie and Nouvelle-Aquitaine have in common elements that have enabled them to develop and institutionalize the practice of HIA in their respective regions; namely, a strong will on the part of public health organizations to perform HIAs and a strong culture of commitment to intersectoral action with municipal partners. However, the two regions have made opposing choices concerning the conducting of HIAs. In Montérégie, we are witnessing the complete management of the HIA process by the PHD, while in Nouvelle-Aquitaine, the strategy implemented is based on a distribution of responsibilities among several organizations. As has been observed, the concentration of roles in the Montérégie region leads to greater consistency of action as concerns the conducting of HIAs and promotes greater skills development among professionals in the PHD. On the other hand, in Nouvelle-Aquitaine, even though the system increases the potential for innovation through the multiplication of actors, the dispersion of responsibilities has been identified as a factor that weakens the leadership required for the proper execution of an HIA approach.

These different implementation options are explained by different regional and even national contexts. While in Montérégie the initial aspiration was to support the development of healthy public policies through HIA, the motivations in Nouvelle-Aquitaine relate more to a desire to decentralize public health services by devolving responsibility to the municipalities. The comparison of the experiences of Montérégie and Nouvelle-Aquitaine shows once again that HIA can be anchored in very different organizational and administrative contexts. The experiences reported here demonstrate the flexibility of the HIA approach and its adaptability to a multitude of situations. It also emerges from the comparative analysis that the success of implementing an HIA approach is largely dependent on the willingness of public health authorities to get involved professionally or through funding. Finally, the strong leadership of public health organizations helps to maintain the focus on the objective of supporting municipal decision-makers in adopting healthier public policy.

## Figures and Tables

**Table 1:** Main features of the HIA implementation models in Montérégie and Nouvelle-Aquitaine.

Analytical Dimensions	Montérégie	Nouvelle-Aquitaine
**A. Contextual factors triggering HIA**		
**Political support and commitment**	Commitment to healthy public policy	Commitment to healthy public policy
	HIA pilot project in 2007–2008	3 HIA pilot projects launched in 2015
**Policy or administrative framework**	HIA included in the regional PH action plan in 2009	HIA included in the REHP 2017–2021
		HUP frameworks
**Legal mandate**	Not mandatory, conducted voluntarily, notwithstanding section 54 at the national level	Not mandatory, conducted voluntarily
**B. Implementation scheme**		
**Stewardship** (lead agency providing framework, support and supervision)	PHD	Environmental Health Department at the RHA
	Knowledge broker	RAHPE
**Roles and responsibilities**	Regional public health teams: scoping, analysis, recommendations	RHA and RAHPE territorial agents, Municipalities: selection and establishment of ToR
	Local public health teams and Municipalities: screening, recommendations	Regional Health Observatory (RHO), private firms, consultants: analysis, recommendations
**Technical support and expertise**	Scientific committee (professionals from the PHD)	Technical committee (RHA, RAHPE, HIA experts)
**HIA model**	Broad model of health Stand-alone	Broad model of healthStand-alone
	Decision-support HIAs	Decision-support HIAs
**C. HIA delivery**		
**Proponents**	Municipalities	Municipalities
**Practitioners’ profile and capacities**	Public health professionals	RHO, private firms and consultants. Mainly trained on the job (basic training + learning by doing approach)
	Resource persons	
	Learning by doing approach	
**Type of HIA**	Intermediate	Mostly intermediate HIAs
**Decision level**	Municipal projects	Municipal projects
**Sector**	Urban planning and social policy	Mainly housing and urban planning
**Funding**	Sustained budget within the general budget of the Public	Dedicated funds within the 2017–2021 REHP (allocated to RAHPE and municipalities commissioning HIAs)
	Health Department (1 position, working hours of scientific committee members)	
**Capacity building**	Ongoing training of new professionals joining the regional HIA team	1-day training targeting all stakeholders at the scoping stage
**Resource generation** (practice framework, methods, tools, evidence)	Tools developed by the regional HIA team	HIA website (ToR, screening and advocacy tools, HIA reports)

HUP: Healthy Urban Planning, PHD: Public health department, RAHPE: Regional Association for Health, Promotion and Education, REHP: Regional Environmental Health Program, RHO: Regional Health Observatory, and ToR: Terms of Reference.
